# Obsessive-compulsive disorder is characterized by decreased Pavlovian influence on instrumental behavior

**DOI:** 10.1371/journal.pcbi.1009945

**Published:** 2022-10-10

**Authors:** Ziwen Peng, Luning He, Rongzhen Wen, Tom Verguts, Carol A. Seger, Qi Chen

**Affiliations:** 1 Key Laboratory of Brain, Cognition and Education Sciences, Ministry of Education, Guangzhou, China; 2 School of Psychology, Center for Studies of Psychological Application, and Guangdong Key Laboratory of Mental Health and Cognitive Science, South China Normal University, Guangzhou, China; 3 Department of Child Psychiatry, Shenzhen Kangning Hospital, Shenzhen University School of Medicine, Shenzhen, China; 4 Department of Experimental Psychology, Ghent University, Ghent, Belgium; 5 Department of Psychology, Colorado State University, Colorado, United States of America; University College London, UNITED KINGDOM

## Abstract

Obsessive-compulsive disorder (OCD) is characterized by uncontrollable repetitive actions thought to rely on abnormalities within fundamental instrumental learning systems. We investigated cognitive and computational mechanisms underlying Pavlovian biases on instrumental behavior in both clinical OCD patients and healthy controls using a Pavlovian-Instrumental Transfer (PIT) task. PIT is typically evidenced by increased responding in the presence of a positive (previously rewarded) Pavlovian cue, and reduced responding in the presence of a negative cue. Thirty OCD patients and thirty-one healthy controls completed the Pavlovian Instrumental Transfer test, which included instrumental training, Pavlovian training for positive, negative and neutral cues, and a PIT phase in which participants performed the instrumental task in the presence of the Pavlovian cues. Modified Rescorla-Wagner models were fitted to trial-by-trial data of participants to estimate underlying computational mechanism and quantify individual differences during training and transfer stages. Bayesian hierarchical methods were used to estimate free parameters and compare the models. Behavioral and computational results indicated a weaker Pavlovian influence on instrumental behavior in OCD patients than in HC, especially for negative Pavlovian cues. Our results contrast with the increased PIT effects reported for another set of disorders characterized by compulsivity, substance use disorders, in which PIT is enhanced. A possible reason for the reduced PIT in OCD may be impairment in using the contextual information provided by the cues to appropriately adjust behavior, especially when inhibiting responding when a negative cue is present. This study provides deeper insight into our understanding of deficits in OCD from the perspective of Pavlovian influences on instrumental behavior and may have implications for OCD treatment modalities focused on reducing compulsive behaviors.

## Author summary

One of the salient symptoms of OCD is compulsive behavior that is difficult to control or suppress. Neuropsychological studies indicate that people with OCD tend to engage in habitual behaviors rather than flexibly adjusting behaviors according to their goals. Behavior can also be affected by cues learned via a separate Pavlovian system that are associated with reward or punishment. Positive Pavlovian cues previously associated with reward typically increase vigorous active engagement whereas negative Pavlovian cues previously associated with punishment typically decrease engagement. In this study, behavioral experiments and computational modeling were used to investigate the effect of Pavlovian cues on instrumental behavior in both clinical OCD patients and healthy controls. People with OCD were less affected by the Pavlovian cues than healthy controls when performing a previously learned instrumental task, especially when negative cues associated with punishment were present.

## Introduction

Obsessive-compulsive disorder (OCD) is characterized by excessive and life-disrupting intrusive thoughts (obsessions) or irresistible behavioral urges (compulsions) [1]. Compulsivity results in the (sometimes stereotyped) repetition of actions that do not produce valuable outcomes [2]. Though OCD patients report that they are aware that their compulsive behaviors are excessive and ineffective, they are unable to inhibit these instrumental behaviors. Pavlovian cues (associations between and rewarding or punishing outcomes learned via classical conditioning mechanisms) can affect instrumental behavior through Pavlovian-instrumental transfer (PIT)[3–10]. PIT has been widely studied in substance use disorders, which are also characterized by the transdiagnostic property of compulsivity[11,12]. However, we know little about the role of the Pavlovian system and how it interacts with instrumental learning in OCD patients. Therefore, our goal in this study was to test whether and how Pavlovian cues affect instrumental behavior in OCD using a standard test of PIT.

Instrumental behavior is affected by the Pavlovian system through the mechanism of Pavlovian-to-instrumental transfer. A classic paradigm that has been used to study the effect of Pavlovian conditioned stimuli (CSs) on instrumental behavior is the Pavlovian-Instrumental Transfer (PIT) task. It has been widely used in both nonhuman animals[13] and humans[14,15]. PIT includes two training stages and one transfer stage[3,7,16]. In the instrumental training stage participants learn instrumental behaviors (typically associated with instrumental cues) to gain reward or avoid punishment. In the Pavlovian training stage, participants learn the association of Pavlovian cues (which are different from the instrumental cues) with reward or punishment. After training, in the Pavlovian-instrumental transfer stage, participants perform the instrumental behavior in the presence of both instrumental and Pavlovian cues. PIT is defined as an increase in responding or response vigor in the presence of positive Pavlovian cues, and a decreased in responding or response vigor in the presence of negative Pavlovian cues[14,17,18].

Transfer further depends on the nature of the outcomes. In specific PIT, Pavlovian stimuli enhance performance selectively for the specific outcome that the cue predicts[13,19]. For example, a Pavlovian cue associated with money may increase instrumental behavior to earn money. In general PIT, Pavlovian stimuli have a more general motivating effect that extends across different outcomes. For example, in general PIT a Pavlovian cue associated with money may enhance an instrumental behavior to obtain food[13,20]. At times the instrumental and Pavlovian associations can lead to conflict: for example, in Go/No-Go tasks subjects mistakenly take actions when they should instead withhold acting in face of reward-predictive stimuli, and they withhold their actions when they should respond to punishment-predictive stimuli [17,21].

The dominant associative learning theory of PIT is the Stimulus-Outcome-Response theory (S-O-R) [14,22]. S-O-R theory states that during instrumental training subjects learn outcome-response associations in parallel with the response-outcome associations that are involved in behavioral control. The Pavlovian system controls valence-dependent behaviors through stimuli-outcome associations [18,21,23]. In the S-O-R theory these Pavlovian stimulus-outcome associations interact with the instrumental outcome-response associations, promoting approach and engagement in presence of reward and inhibition or active escape in presence of punishment [5,6,8,9,17,21,24]. More recently some researchers have proposed an alternative theory of specific PIT, arguing that it relies on declarative knowledge of the Pavlovian contingencies that then affect goal-directed behaviors, particularly in humans [10,25,26]. This theory postulates that participants use the Pavlovian cues as contextual indicators of the value and presence of rewarding outcomes, which then affect their goal-directed choice to increase responding and/or effort accordingly [25]. Evidence for this view includes that many studies find that PIT is sensitive to outcome devaluation, an indicator of goal-directed control, and human participants typically have verbalizable knowledge of the relationship between the Pavlovian cue and their increased responding [10,25].

A disruption in how Pavlovian responses affect instrumental behaviors is thought to play a key role in many psychiatric syndromes [5,7,27]. For example, the environmental cues associated with experienced drug intake could trigger instrumental drug-seeking through Pavlovian conditioning among individuals with substance use disorders (SUDs)[28].PIT is increased in patients with SUDs, and the magnitude of PIT has been shown to predict likelihood of relapse [7,29,30], consistent with a causal role for reward cues in compulsive drug-taking behavior. These results are consistent with an incentive salience theory of addictions: cues related to substances of abuse acquire independent incentive salience via Pavlovian associative learning, and this incentive salience then serves to reinforce the development of compulsive drug taking[31,32]. Although PIT is increased in SUDs, a study of patients with schizophrenia reported decreased PIT in comparison with healthy controls[33].

It has been theorized that incentive salience also plays a role in OCD in that compulsion and obsession-related cues (e.g., cleanliness cues) can reinforce the development of compulsions (e.g., compulsive hand washing) through association with relief from anxiety [34]. Previous research has not examined PIT in a clinical group of OCD patients; however, one previous study examined high and low levels of compulsive behaviors in a non-clinical sample and found that in this situation the high compulsive group showed less PIT [4]. The aim of our study was to investigate effects of Pavlovian cues on instrumental behavior in both clinical OCD patients and healthy controls. We utilized a standard PIT task with separate instrumental, Pavlovian, and PIT stages. We also used reinforcement learning models to identify learning parameters from the instrumental and PIT stages that might elucidate the mechanisms that underlie differences in PIT.

## Method

### Ethics statement

The study was approved by the Human Research Ethics Committee for Non-Clinical Faculties of the School of Psychology, South China Normal University. All methods were performed according to their relevant regulations and guidelines. All participants provided written informed consent for the study and were told that their payment was dependent on how they performed in the experiment (i.e., basic compensation of 30 yuan plus a bonus of 0 to 5 yuan).

### Participants and procedure

Patients were recruited via referral from psychiatric experts at Shenzhen Kangning Hospital in the city of Shenzhen. Controls were recruited via online advertisement. OCD was diagnosed by two fully certified consultant psychiatrists according to DSM-5 criteria using an extended clinical interview and supplemented with the Mini International Neuropsychiatric Interview[35]. Seven OCD patients had comorbid depression, two patients had comorbid anxiety disorder and two patients had comorbid bipolar disorder. All participants were asked to complete the Yale-Brown Obsessive-Compulsive Scale (Y-BOCS) [36], the Beck Depression Inventory (BDI) [37], and the State-Trait Anxiety Inventory (STAI) [38].

Exclusion criteria were being younger than 15 or older than 50, a past or current neurological disorder, substance abuse/dependence, and/or endocrine and cardiac disorders. HC participants were excluded from the study if they had a past or current psychiatric disorder, and OCD patients were excluded if they were diagnosed with a psychiatric disorder other than the disorders mentioned above.

A total of 32 OCD patients and 31 healthy controls (HC) with normal or corrected to normal vision were tested on the task. Two OCD patients were excluded from the behavioral analyses because of failure to finish the experiment or poor performance in the instrumental stage, and two more OCD patients were excluded from any analysis involving scales due to the failure of data collection. OCD patients and HC were matched in sex, age, and education. Of the 30 patients, 25 were medicated and 5 were not medicated at the time of the study (See Table 1). Some of the patients were taking more than one medication. Demographic, clinical, and medication data are displayed in Table 1.

**Table 1:** Characteristics of the participants.

	HC mean (SD)	OCD mean (SD)	P
**Demographic**			
Male (%)	65%	70%	0.655
Age	27.23 (9.09)	25.70 (7.04)	0.468
Education	13.32 (2.89)	11.93 (3.04)	0.072
**Clinical**			
Y-BOCS obsessions	3.11 (3.82)	10.72 (3.56)	<0.001
Y-BOCS compulsions	2.13 (2.78)	9.07 (4.45)	<0.001
Y-BOCS total	5.24 (5.64)	19.79 (7.60)	<0.001
STAI-State	34.83 (7.03)	52.71 (15.73)	<0.001
STAI-Trait	39.87 (7.85)	55.96 (13.62)	<0.001
STAI-Total	73.93 (12.45)	101.43 (38.69)	0.002
BDI	6.90 (7.03)	23.36 (13.06)	<0.001
**Medication**			
SSRI		n = 21	
BZDs		n = 10	
Non-BZDs		n = 4	
AAs		n = 6	
AED		n = 3	
TCAs		n = 2	
SSNRIs		n = 1	
Antimanic drugs		n = 1	

Some participants were taking more than one medication. Education: Total years of education beginning from primary school excluding kindergarten; Y-BOCS, Yale-Brown Obsessive-Compulsive Scale; STAI, State-Trait Anxiety Inventory; BDI, Beck Depression Inventory-II; SSRI: Selective serotonin reuptake inhibitors; BZDs: Benzimidazole; Non-BZDs: Non-Benzimidazole; AAs: Atypical antipsychotics; AED: Antiepileptic drugs; TCAs: Tricyclic anti-depressive drugs; SSNRIs: Selective serotonin-norepinephrine reuptake inhibitors.

### Pavlovian-instrumental transfer task

**Fig 1:**
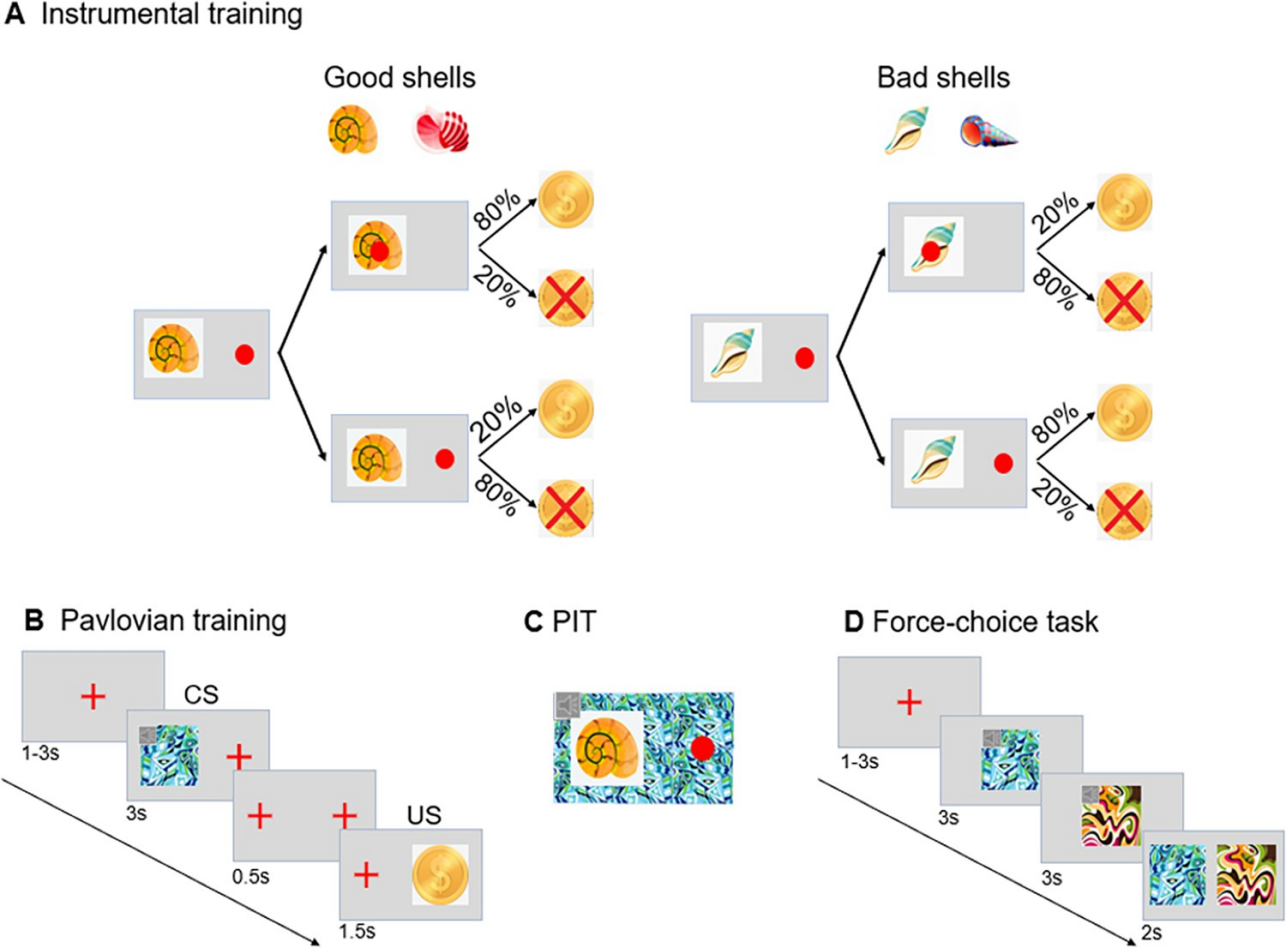
PIT paradigm. (A) Instrumental training. Participants were instructed to learn to classify good shells and bad shells via trial and error with feedback. Participants responded by repeatedly pressing the Enter key. Each press moved the red dot incrementally closer to the center of the shell. A total of 6 or more keypresses would move the red dot into the center of a shell and meet the criterion for collecting the shell. Collecting a good shell resulted in an 80% chance of receiving a reward (indicated by the coin image) and a 20% chance of punishment (indicated by the coin with a red cross superimposed image); contingencies were reversed for bad shells. (B) Pavlovian training. Participants were asked to remember the CS-US pairings. The CS consisted of simultaneous visual and auditory stimuli. The US+ (positive) was an image of a coin, the US- (negative) was an image of a coin with a red cross superimposed, and the US0 (neutral) was a blank screen. (C) Pavlovian-to-instrumental transfer. This stage was designed to test the influence of CSs on instrumental conditioning. Participants performed the same task as in A, but with simultaneous presentation of a CS, and without explicit indication of the reward obtained. (D) Forced-choice task. Participants viewed each stimulus individually with the auditory component present, and then viewed the visual stimuli alone side by side. They were instructed to choose the CS with a better outcome.

The task was modified from that developed by[39]. Every participant completed the subtasks in the following order: (a) Stage 1: Instrumental training; (b) Stage 2: Pavlovian training; (c) Stage 3: PIT; and (d) Stage 4: Forced-choice task (Fig 1).

In the instrumental training stage, participants were required to collect shells to obtain as many coins as possible. There were 4 shells (instrumental stimuli, IS) including two good shells (referred to as G1 and G2) and two bad shells (referred to as B1 and B2) (See Fig 1A). Visual features of all four shells were highly discriminable. Participants responded by pressing the Enter key. Each press moved the red dot incrementally closer to the center of the shell. A total of 6 or more keypresses would move the red dot into the center of a shell and result in collecting the shell. If the number of presses was less than 6, the shell was considered to not have been collected. In trials with good shells, participants were rewarded for 80% of collection actions performed, and 20% of no collection actions, whereas in trials with bad shells, 80% of no collection actions and 20% of collection actions were rewarded. Trials that were not rewarded received punishment (loss of 1 coin). Each instrumental training trial started with a red fixation cross (+) presented for a randomly determined interval in the range of 1–3 s. Then, a shell appeared on the right or left side of the screen randomly with the same number of occurrences. Participants had 2 s to decide whether to collect the shell and execute the required keypresses. Feedback followed, consisting of a 1.5 s presentation of coin (reward) or a coin with a superimposed red cross representing the loss of a coin (punishment) (Fig 1A). Participants completed a total of 3 blocks of trials of unequal length. The first two blocks each contained 20 trials and trained; here, participants were trained on only two of the stimuli, G1 and B1 in block 1, and G2 and B2 in block 2. In the final block all four stimuli were presented in random order. This design was chosen because in pilot studies participants found it difficult to learn all four shells simultaneously. In the final block participants continued until they either met a criterion of 80% correct choices over 10 trials or completed a total of 40 trials.

Pavlovian training began with a red fixation appearing on the screen for a randomly determined interval in the range of 1–3 s followed by the conditioned stimulus (CS) on the right or left side of the screen. The CS consisted of a visual and auditory component and was presented for 3s. The visual components of the CSs were abstract fractal images (see Fig 1 for examples). The three different audio components for the Pavlovian stimuli were a repeated “beeping” noise, a repeated “tick-tock” noise, and a repeated “gear” noise. 500ms after the offset of the CS, participants were presented with the positive, negative or neutral unconditioned stimulus (US) associated with the CS on the opposite side of the screen for 1500ms. A positive CS (CS+) was followed by a reward, which was indicated by a coin. A negative CS (CS-) was followed by a coin with a superimposed red cross. A neutral CS (CS0) was followed by nothing indicated by a blank screen. The CS-US pairings were fixed and deterministic within and counterbalanced between participants. Participants were instructed to carefully observe and memorize the CS-US associations. All participants completed 24 trials (Fig 1B), with each CS-US (CS+, CS-, CS0) pair presented 8 times.

In the PIT stage, participants were told that this stage was similar to the instrumental training stage, and they were required to try their best to obtain coins by following the rules they had learned in the first two stages. This stage was identical to the instrumental training, except for the following: (a) task-irrelevant CSs identical to those in the Pavlovian training stage (both visual and auditory components) appeared as background (see Fig 1C), and (b) the whole task was completed in extinction without feedback to avoid confounding effects of new learning. However, participants were told that their choices still counted toward the final monetary outcome. After the presentation of a fixation cross for a randomly determined interval in the range of 1–3 s, one of the four shells appeared on the right or left side of the screen for 3 s during which participants could choose to collect or not and execute the keypresses required. The task consisted of 72 trials, with each combination of instrumental stimulus and Pavlovian stimulus (IS-CS) appearing equal times (Fig 1C).

In a final (forced choice) stage (Fig 1D), to verify the acquisition of CS-US associations, participants were presented with pairs of CSs and instructed to choose the better CS of the two. All possible combinations of two CSs were presented in an interleaved, randomized order 3 times. Each CS of one CSs pairing was first presented alone for 3s during which the auditory component of the CS was included. Then the two visual components of the CSs were presented together for 2s on opposite sides of the screen and participants were asked to choose the better of the two CSs by pressing the corresponding left or right side key on the computer keyboard. If participants failed to perform better than chance, they were excluded for further analyses. All participants met this criterion and were included in the analyses.

### Computational models

We used modified Rescorla-Wagner (RW) models to capture the behavioral choices and cognitive process using methods similar to those in a previous study[8]. The model space consisted of 6 models for the instrumental training stage and 3 models for the PIT stage.

In the instrumental training stage, there were two instrumental stimuli *S*^*I*^ (i.e., good or bad shell. Both good shells were treated as identical stimuli and assigned the same parameters; the same procedure was followed for the bad shells) and two possible actions *a* (i.e., collect or refrain from collecting). At each trial *t*, the instrumental weight $W_{t}^{I}$ of action *a* in presence of an instrumental stimulus *S*^*I*^ consisted of a) the expected instrumental value of that stimulus-action pair *Q*_*t*_(*S*^*I*^, *a*) and b) a fixed bias for collect actions *b*(*a*). The probability to pick action *a* was generated using a softmax-based action selector:

$$W_{t}^{I}(S^{I},a)=Q_{t}(S^{I},a)+b(a))$$

$$p_{t}(a=collect|S^{I})=\frac{exp(W_{t}^{I}(S^{I},collect))}{\exp\left(W_{t}^{I}(S^{I},collect)\right)+exp(W_{t}^{I}(S^{I},refrain))}$$ (1)

$$=\frac{1}{1+exp{(-(W}_{t}^{I}(S^{I},collect)-W_{t}^{I}(S^{I},refrain))}$$ (2)

As the softmax-based action selector was used, it was the difference of $W_{t}^{I}$ between collect and refrain actions that was critical for generating the probability of the action to be taken. The bias for refraining from collection was always zero to ensure the parameters would be identifiable and not be redundant. If instead the bias for the collect action was set to zero, the results of parameter estimation revealed exactly the same values but oppositely signed. The expected instrumental values *Q*_*t*_(*S*^*I*^, *a*) were updated according to a RW-like rule with two fixed learning rates *ϵ* after receiving either reward feedback or punishment feedback *r*_*t*_∈{−1, 1}. The subjective value of the feedback delivered in the experiment may vary across subjects, so the sensitivity parameter *ρ* was added to measure this effect:

$$Q_{t+1}(S^{I},a)=Q_{t}(S^{I},a)+\epsilon (\rho r_{t}-Q_{t}(S^{I},a))$$ (3)

$$\epsilon =\{\begin{array}{c} \epsilon_{\mathrm{pun}},\;r_{t}<0\\ \epsilon_{\mathrm{rew}},\;r_{t}>0 \end{array}$$ (4)

The model described above was the winning model (Model 4) in this study which yielded the largest log-model evidence (defined below) compared with other alternative control models (See Table 2). 6 alternative models were used to fit the behavioral data and compared in the instrumental stage (See Table 2, Model 1–6). Model 1 and Model 2 assumed an identical learning rate and identical sensitivity for positive and negative feedback. Model 3 and Model 4 assumed separate learning rates for reward and punishment feedback with identical sensitivity. Model 5 and Model 6 assumed identical learning rates but separate sensitivities for positive and negative feedback:

$$\rho =\{\begin{array}{c} \rho_{\mathrm{pun}},\;r_{t}<0\\ \rho_{\mathrm{rew}},\;r_{t}>0 \end{array}$$ (5)

**Table 2:** Parameters contained in the models and Bayesian model comparison.

	Relative LME (N = 61)	Parameter
M1	-137.336	*ϵ*, *ρ*
M2	-55.488	*ϵ*, *ρ*, *b*(*a*)
M3	-23.048	*ϵ*_rew_, *ϵ*_pun_, *ρ*
M4	0	*ϵ*_rew_, *ϵ*_pun_, *ρ*, *b*(*a*)
M5	-45.372	*ϵ*, *ρ*_rew_, *ρ*_pun_
M6	-40.286	*ϵ*, *ρ*_rew_, *ρ*_pun_, *b*(*a*)
M4+M7	-76.216	*f* (*S*^*P*^, *a*)
M4+M8	-51.512	*f* (*S*^*P*^, *a*), α
M4+M9	0	*f* (*S*^*P*^, *a*), *η*(*S*^*I*^, *a*)

Model 1, Model 3, and Model 5 assumed no bias for collect actions; in these models, we always set to zero, *b*(*a*_*t*_) = 0, whereas Model 2, Model 4, and Model 6 allow for bias to be estimated.

Models 1–6 were fitted on data in the instrumental task; remaining models were fitted to the PIT task. Each column includes the relative log-model evidence for the model. Higher values (closer to zero) indicate more evidence for the model. The parameters column indicates which parameters were included in the model. *ϵ*, *ϵ*_rew_, and *ϵ*_pun_: single learning rate, learning rate for reward, and learning rate for punishment, respectively. *ρ*, *ρ*_rew_, and *ρ*_pun_: single sensitivity, sensitivity for reward, and sensitivity for punishment, respectively. *b*(*a*): response bias. *f* (*S*^*P*^, *a*): Pavlovian factors. *α*: decay rate for transfer. *η* (*S*^*I*^, *a*): noise for transfer.

Because there were no behavioral measures collected during the Pavlovian stage, no model could be fitted for this stage. To fit choice data in the PIT stage, both instrumental and Pavlovian influences were considered as components that influence choice of actions. The instrumental component was transferred from the instrumental training stage with noise or decay. The Pavlovian influences were treated as free parameters to be estimated by fitting trial-by-trial behavioral choices. These free parameters quantified how strongly the particular Pavlovian stimuli influence the instrumental behavior (i.e., promoting the action of collecting), which depends on the associated outcome learned in the Pavlovian training stage.

The instrumental component was quantified by action weights fitted at the end of the instrumental stage with the addition of noise defined by the potential mechanism of generalization. The Pavlovian factors *f* (*S*^*P*^, *a*) for a particular Pavlovian stimulus- action pair were constant free parameters and were estimated as for each of the three Pavlovian stimuli (i.e., positive, negative, and neutral Pavlovian stimuli). If the estimated Pavlovian factor was larger (smaller) than zero, the corresponding Pavlovian stimulus increased (inhibited) the collection action. The Pavlovian factors for the refrain action *f*(*S*^*P*^, *refrain*) were always set to be zero following the study of Huys et al. [8]. Therefore, the estimated Pavlovian factors captured the individual-specific strength of Pavlovian influence on the action of collecting in the PIT stage. Adding Pavlovian factors and the instrumental action weight $W_{T}^{I}(S^{I},a)$ learned at the end (i.e., trial *T*) of the instrumental training stage results in action weights $W_{t}^{PIT}(S^{I},S^{P},a)$ in the presence of instrumental stimulus *S*^*I*^ and Pavlovian stimulus *S*^*P*^ in the PIT stage:

$$W_{t}^{PIT}(S^{I},S^{P},a)=W_{IT}(S^{I},a)+f(S^{P},a)$$ (6)

A softmax function similar to Eq (2) was combined with action weights in the PIT stage to generate the probability of each action.

Similar to the previous study[8], we tested 3 potential mechanisms of generalization (i.e., the transformation of learned expected instrumental values *Q*_*T*_(*S*^*I*^, *a*) from the end of the instrumental stage to the PIT stage) (See Table 2, Model 7–9). Model 7 assumes that the expected instrumental values *Q*_*T*_(*S*^*I*^, *a*) at the end of the instrumental stage are transferred to the PIT stage without any loss. Model 8 allows for exponential decay of expected instrumental values *Q*_*T*_(*S*^*I*^, *a*) over time by including a free parameter *α*, 0≤*α*≤1:

$$Q_{t+1}(S^{I},a)=\alpha Q_{t}(S^{I},a)$$ (7)

Model 9 assumes generalization with a fixed noise *η*(*S*^*I*^, *a*), which is sampled from the corresponding prior Gaussian distribution of each instrumental stimulus. The prior Gaussian distributions (i.e., group distribution) of a particular instrumental stimulus was the same for everyone, and its mean and variance estimated were estimated with individual noise parameters (see Model fitting). So that the action weights in the PIT stage in presence of particular instrumental and Pavlovian stimuli were the linear combination of the action weight learned in the instrumental stage, the Pavlovian factor, and the generalization noise:

$$W^{PIT}(S^{I},S^{P},a)=W_{IT}(S^{I},a)+f(S^{P},a)+\eta (S^{I},a)$$ (8)

The noise for the refrain from collection action was set to be zero (akin to the bias and Pavlovian factors).

### Model fitting

A hierarchical Bayesian method was used to estimate the parameters of the models by fitting the trial-by-trial behavioral data [8,40,41]. Parameters for each participant were estimated using the maximum a posteriori method with a prior which was estimated from the distribution of group parameters to avoid overfitting. There are multiple ways to set a prior in studies involving more than one group [42]. We did not use an empirical prior or separate priors for each group in order to minimize the chance that the correlation between parameters and the severity of symptoms would be overestimated [42]. In our study, data for the HC and OCD groups were estimated using the same uninformed group prior.

This hierarchical Bayesian method was performed in two steps and the individual and group parameters were updated in an iterative way. In the first step, the maximum a posteriori (MAP) method was used to estimate free parameters for every participant. In this step, a wide initial Gaussian prior distribution (with a mean of 0 and a variance of 10) was chosen as the group-level distribution of all individual parameters including both HC and OCD. A nonlinear derivative-based optimization algorithm (as implemented in the fminunc routine in MATLAB, MathWorks) was used for the maximum a posteriori estimation for each participant separately. The prior was chosen to enable parameters to vary across a wide range to avoid bias. To overcome the problem of local optima, the optimization was carried out multiple times to select the optimal parameter set.

In the second step, the group-level prior parameters were estimated. The group parameters were estimated with the maximum likelihood method given choice data of all subjects. This maximum likelihood was optimized by the expectation-maximization (EM) algorithm with the individual parameters as the latent variables. The Laplace approximation (i.e., assuming a Gaussian distribution as the group-level distribution) was used in the E-step to obtain the expectation of log-likelihood. Then the prior in the first step was taken place by the distribution of parameters across all the participants to re-estimate parameters of each participant. Additionally, the group parameters were also updated by re-estimated individual parameters. Both individual and group parameters were estimated iteratively till convergence. For details of this model fitting procedure, please refer to the study of Huys et al [8].

### Model comparison

To test which model within the model space best captures the behavioral data, we assessed log-model evidence (LME) using the Laplace approximation and the Bayesian information criterion to balance between flexibility and complexity[40,41]:

$$LME=\sum_{n}logP(D^{n}|\theta^{n})+\sum_{n}logN(\theta^{n}|\Theta ,\Sigma )+\frac{1}{2}dNlog2\pi -\frac{1}{2}\sum_{n}\log\left|H_{n}\right|-dlogN$$ (9)

where *D*^*n*^ is the choice data of the *n*th participant, *θ*^*n*^ is the MAP parameter estimate for the *n*th participant, Θ and Σ is the mean and variance of the group distribution estimated by maximum likelihood method, *d* is the number of free parameters of the model, *N* in the second term refers to a multivariate normal distribution with Θ and Σ as the mean and the covariance matrix, *N* in other terms is the number of participants, and |*H*_*n*_| is the determinant of the Hessian matrix of the log-posterior function at *θ*^*n*^. The LME was approximated by the Laplace approximation with a penalty of 2*d* (mean and variance together) for both individual and group parameters to avoid overfitting. For computer code used to implement the model fitting and model comparison procedures, please refer to [40].

### Statistical analysis

At the behavioral level, performance during instrumental conditioning was tested and compared. First, we analyzed total number of trials achieve the learning criterion on the instrumental task (accuracy of 80% over 10 trials in block 3). Second, we analyzed the accuracy of collecting good shells and refraining from collecting bad shells, and the probabilities for stay after reward (i.e., repeat the previous behavior) or switch after punishment (i.e., shift to another behavior) during the instrumental task. For the PIT task, to quantify the magnitude of the PIT effect, the mean number of keypresses (i.e., the average number of keypresses in presence of each Pavlovian stimuli across good and bad shells) were regressed linearly for each CS (CS+, CS0, and CS- were encoded as 1, 0, and -1 correspondingly). To compare the effect of Pavlovian stimuli on instrumental behaviors, the number of keypresses and the probability of collecting (i.e., the percentage of trials in which the subject pressed 6 or more times in all trials of each Pavlovian condition across good and bad shells) were analyzed using mixed effects ANOVAs. All the post hoc t tests were Bonferroni corrected.

At the computational level, estimated free parameters including learning rates, response bias, sensitivity, and expected instrumental values of each stimuli-action pair in the instrumental learning stage as well as the Pavlovian factors in the PIT stage were compared between groups using t-tests and ANOVA. Correlation analyses of scores on Y-BOCS and parameters estimated across all participants from both groups was performed using partial correlation controlling for scores on STAI and BDI. In order to test whether our primary findings were sensitive to depression and anxiety, we also performed ANCOVA with the Pavlovian factors including the scores on STAI and BDI as covariates.

All code and data for reproducing the analyses and figures is available at: https://osf.io/rc2gx/

## Results

### Model-free analyses

**Fig 2:**
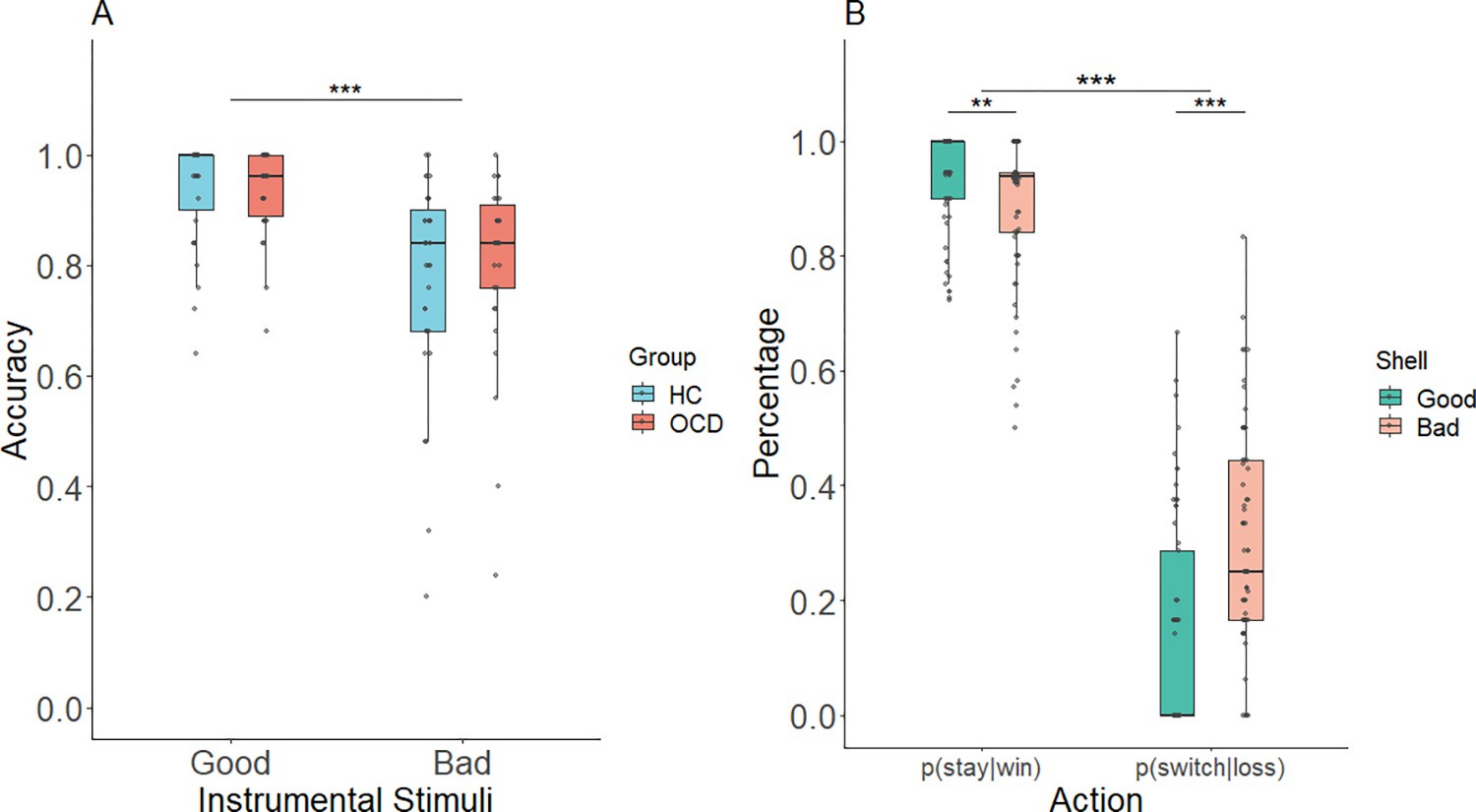
Performance during instrumental training stage. (A) Mean accuracy for good and bad shells (B) The effect of reward and punishment feedback on subsequent behaviors. The graph is collapsed across group as there was no significant group difference. “*”: p < 0.05; “**”: p < 0.01; “***”: p < 0.001.

In the instrumental stage, the analyses revealed that instrumental conditioning did not differ across groups. There were no significant group differences in total number of trials to achieve the learning criterion (accuracy of 80% over a set of 10 trials in block 3) in the two groups (HC = 50.741 (2.503), OCD = 50.600 (2.159), Mann-Whitney U test: *p* = 0.989. Note that means reflect total number of trials including the 40 trials completed in blocks 1 and 2). This pattern of results indicates that participants from both groups were able to learn the instrumental contingencies during the first two blocks of training and required only a minimum number of trials in block 3. As shown in Fig 2A, for the accuracy dependent measure (calculated across all three blocks), performance was above chance level for both stimuli and groups. A 2 (Instrumental stimulus: good and bad shells) × 2 (Group: OCD and HC) mixed effects ANOVA revealed a main effect of the instrumental stimuli, such that participants performed better when collecting good shells than when refraining from collecting bad shells (*F*(1,59) = 40.612, *p <* 0.001) (Fig 2A). There was neither a group difference (*F*(1,59) = 0.239, *p* = 0.627) nor an interaction of group × instrumental stimulus (*F*(1,59) = 0.564, *p* = 0.456) in average accuracy in the instrumental training stage.

The probabilities for stay (i.e., repeat the previous behavior) or switch (i.e., shift to another behavior) on trials following reward or punishment feedback were analyzed to test the respective effects of reward and punishment outcomes on subsequent behavior in the instrumental training stage. A 2 (Group: OCD and HC) × 2 (Instrumental stimulus: good and bad shell) × 2 (Feedback: win-stay and lose-switch) mixed effects ANOVA showed no significant interaction of group × IS × feedback (*F* (1,59) = 0.246, *p =* 0.622) but a significant interaction of IS × feedback (*F* (1,59) = 37.415, *p <* 0.001). Post hoc tests revealed the probability to stay after reward was higher in collecting good shells than refraining from collecting bad shells (*t* = 2.995, *p =* 0.004), which reversed in the probability of switching after punishment (*t* = -5.841, *p <* 0.001) (Fig 2B). The main effect of Feedback (*F* (1,59) = 586.448, *p <* 0.001) showed that the probability of repeating a rewarded action was higher than switching to another action after punishment (*t* = 24.217, *p <* 0.001). The overall switching probability was low, likely because of the probabilistic nature of the task: 64.59% of the punishments were received on the 20% of trials in which punishment followed the normatively correct action. No other effects were found significant (*ps* > 0.100).

**Fig 3:**
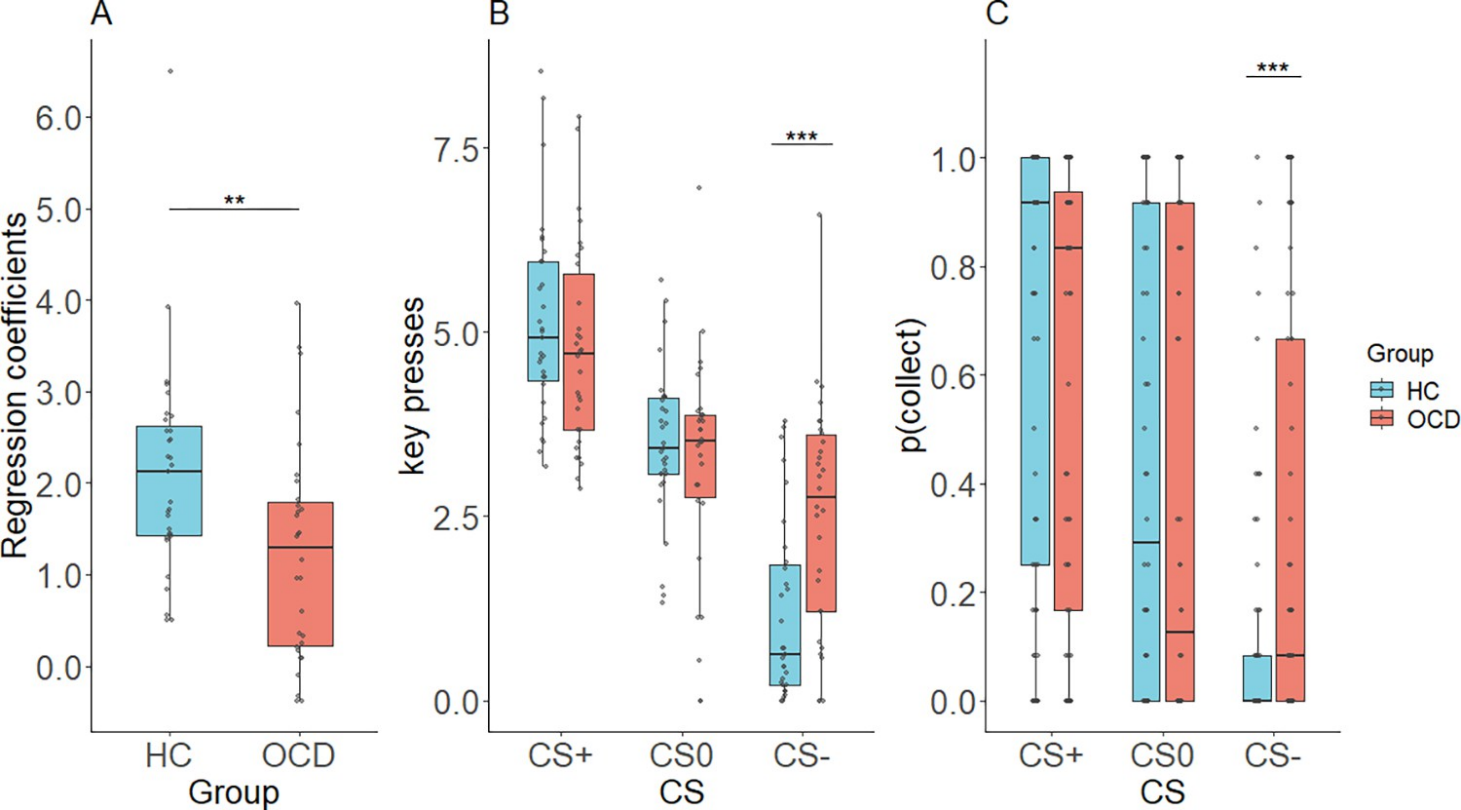
PIT effects during Pavlovian-to-instrumental transfer. (A) The magnitude of the overall PIT effect quantified by linear regression coefficients by regressing each subject’s mean number of key presses onto positive, neutral, and negative CSs. (B) Mean key presses under each Pavlovian condition. (C) Mean probability of collecting under each Pavlovian condition.

In the PIT stage, we tested the effect of Pavlovian stimuli on instrumental behavior by analyzing the average number of keypresses in presence of positive, negative, and neutral Pavlovian stimuli across good and bad shells. The PIT effect we examined included both appetitive (reward) and aversive (punishment) components. Previous research has established that appetitive Pavlovian stimuli elicit vigorous active engagement, whereas aversive Pavlovian stimuli are associated with behavioral inhibition. [21].The overall PIT effect measured by the linear regression coefficients, which included both appetitive and aversive components, was significant (*β* = 1.565, *t* = 12.40, *p <* 0.001). Larger linear regression coefficients were found in HC compared to OCD (*t* = 2.903, *p* = 0.005) showing a stronger PIT effect in HC (Fig 3A).

To compare the different effects of the valences of Pavlovian stimuli, a 2 (Group: OCD and HC) **×** 3 (CS: CS+, CS0, CS-) mixed effects ANOVA was performed with the dependent variable of number of keypresses. The results revealed a main effect of CS (*F*(1.620, 95.608) = 95.569, *p* < 0.001, Greenhouse-Geisser corrected) indicating a significant PIT effect. Post hoc tests showed a significant difference in number of keypresses between all pairs of conditions (CS- < CS0 < CS+, *p*s < 0.001). Moreover, the significant interaction of CS × group (*F*(1.620, 95.608) = 7.790, *p* = 0.002, Greenhouse-Geisser corrected) indicates that the PIT effect was different in OCD patients than in HC. Post hoc tests indicate that the interaction may be driven by different effects of CS- in the two groups, in that OCD made more keypresses in CS- than HC (*t* = 3.790, *p <* 0.001), but showed no differences in keypresses during CS+ (*t* = 0.637, *p* = 0.526) and CS0 (*t* = 1.068, *p* = 0.290) (Fig 3B).

For the dependent variable of probability of collecting the shell, shown in Fig 3C, a mixed-effect CS × group ANOVA revealed a significant main effect of CS (*F*(1.751, 103.321) = 96.868, *p* < 0.001, Greenhouse-Geisser corrected) and significant interaction of CS × group (*F*(1.751, 103.321) = 11.075, *p* < 0.001, Greenhouse-Geisser corrected). Post hoc tests indicated that the significant interaction was driven by performance in the CS- condition, with OCD collecting fewer shells than HC in CS- (*t* = 3.899, *p <* 0.001). Within the HC group there were significant differences in probabilities of collecting between all pairs of conditions such that more positive CS was associated with greater collection probability (CS+ > CS0 > CS-, *p*s < 0.001). Within the OCD group in contrast the CS+ was associated with a significantly greater collection probability than CS0 (*t* = 4.583, *p* < 0.001) and CS- (*t* = 6.164, *p <* 0.001) but there was only a trend toward CS0 showing a greater collection probability than CS- (*t* = 2.438, *p* = 0.053) (Fig 3C). Overall, across both dependent measures these results indicate a lower overall PIT effect in the OCD group which was especially apparent for negative CSs. This pattern of results suggests substantially impaired aversive PIT in OCD, with only mildly (if at all) impaired appetitive PIT in OCD.

In stage 4, the forced-choice test to investigate whether participants had learned the valence of the CSs, all participants preferred higher valued CSs overall (that is, the CS followed by a better outcome in Pavlovian training, evidenced by preferring the CS+ over both CS0 and CS-, and CS0 over CS-). The mean accuracy rates in the OCD and HC groups were 96% and 98%, respectively. There was no significant group difference in accuracy rates (Mann-Whitney U test: *p* = 0.110).

### Model-based analyses

In the instrumental stage, the model space included the 6 models described above and listed in Table 2. Higher values (closer to zero) indicate more evidence in favor of the model. For the instrumental training, the result of Bayesian model comparison revealed that model 4, which included parameters for separate learning rates representing different learning rates for rewards and punishments, a single sensitivity parameter, and a bias toward collect actions (See Table 2), explained the data better than other models. In the PIT stage, the model space consisted of three models in which the winning model from the instrumental state (Model 4) was combined with additional parameters. The best fitting model was Model4+Model9, a model which included noisy generalization.

Free parameters from the winning model (Model 4 in Table 2) were compared between groups to examine the characteristics of learning during the instrumental training phase. There was a significant main effect of feedback on learning rates indicated by a mixed effect 2 (Feedback: reward, punishment) × 2 (Group: HC, OCD) ANOVA (*F*(1, 59) = 289.086, *p* < 0.001), but no significant interaction of group × feedback (*F*(1, 59) = 0.345, *p* = 0. 559) and no main effect of group (*F*(1, 59) = 0.297, *p* = 0. 588). Post hoc tests indicated that learning rates for reward outcomes were larger than those for punishment outcomes (*t* = 17.003, *p <* 0.001), which are consistent with the low switch probabilities after punishment feedback seen the in the model-free analyses. Response bias and sensitivity revealed no differences between groups (response bias: *t* = 1.196, *p =* 0.237, sensitivity: *t* = 0.587, *p* = 0.559).

We compared the expected instrumental values of each stimulus-action pair learned at the end of the instrumental stage with a 2 (Instrumental stimuli: good, bad) × 2 (Action: collect, refrain) × 2 (Group: HC, OCD) ANOVA. The interaction of IS × action ×group was not significant (*F*(1, 59) = 0.029, *p* = 0.866). However, there was a significant interaction of IS× action (*F*(1, 59) = 791.963, *p* < 0.001) showing the expected instrumental value of collect actions was higher than refrain actions for good shells (*t* = 24.235, *p* < .001), but which reversed for bad shells (*t* = -26.710, *p* < .001). The interaction of IS × action on the expected instrumental value revealed participants had learned to collect good shells and refrain from collecting bad shells successfully. But no main effect of group was observed indicating no instrumental stage learning differences between OCD and HC groups (*ps* > 0.579).

**Fig 4:**
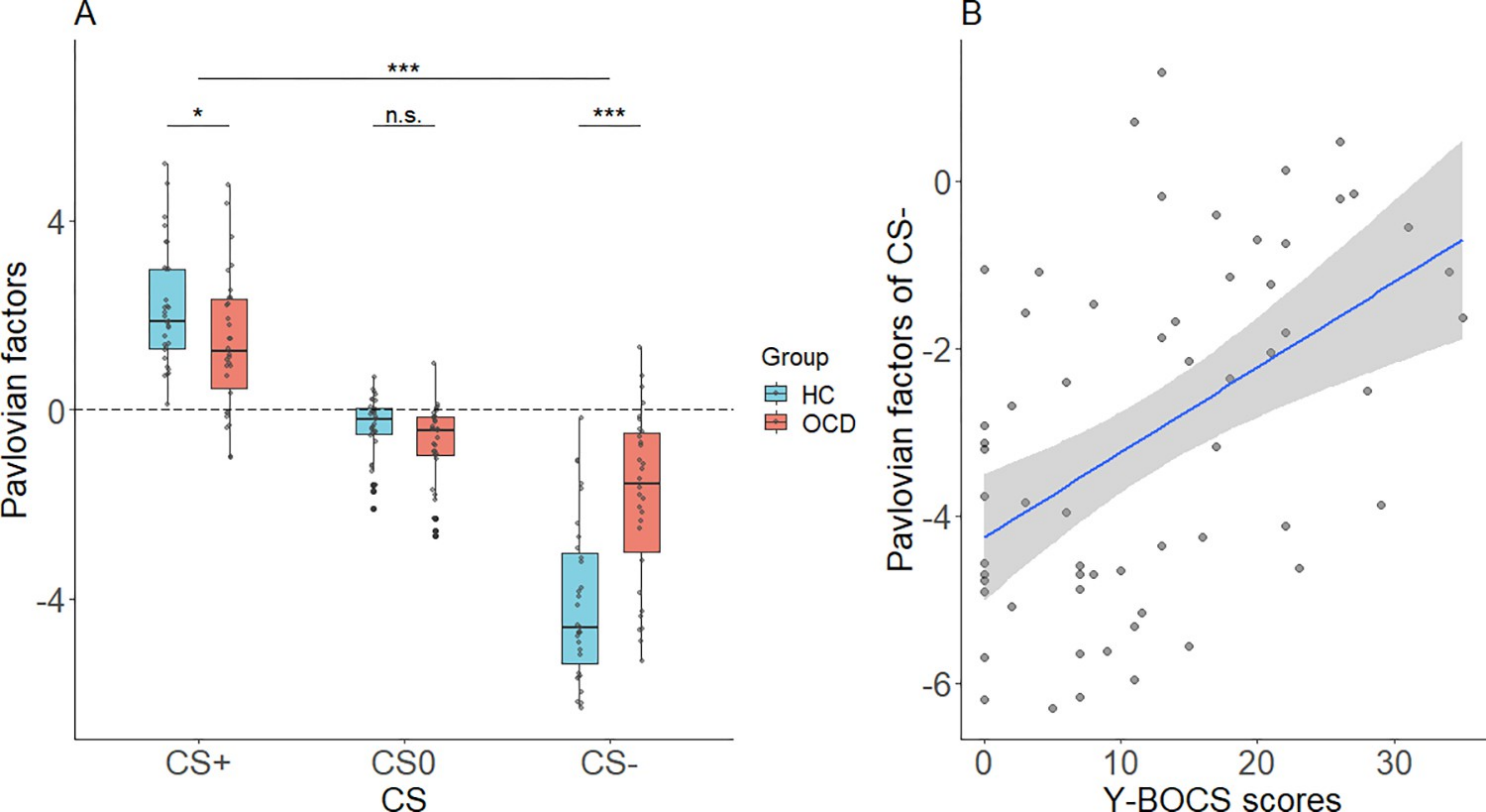
Pavlovian factors in each Pavlovian condition and correlation with Y-BOCS scores. (A) Pavlovian factors in each Pavlovian condition for the OCD and HC groups. The dashed line shows the zero-effect level where Pavlovian factors do not affect instrumental behavior. (B) Y-BOCS scores correlated significantly with Pavlovian factors for CS-.

We next examined parameters from the computational modeling of Pavlovian effects. As described above, we estimated the Pavlovian factors which indicate the strength of the influence of the Pavlovian stimuli on instrumental behavior during the PIT stage of the experiment. Pavlovian factors are normalized and zero-centered so that values greater than zero indicate a positive Pavlovian influence, and values less than zero indicate a negative Pavlovian influence. As shown in Fig 4A, the estimated Pavlovian factor parameters differed across groups. A 2 (Group: HC, OCD) × 3 (CS: CS+, CS0, CS-) ANOVA revealed a significant main effect of CS (*F*(1.642, 96.854) = 152.070, *p* < 0.001, Greenhouse-Geisser corrected) indicating Pavlovian factors across groups were largest for CS+, smaller for CS0, and lowest for CS-(CS+ > CS0 > CS-, *ps <* 0.001) (Fig 4A). A significant group × CS interaction (*F*(1.642, 96.854) = 17.483, *p* < 0.001, Greenhouse-Geisser corrected) revealed in the CS- condition significantly more positive (that is, closer to zero) Pavlovian factors in OCD than HC (*t* = 4.949, *p <* 0.001), which reversed in the CS+ conditions with significantly more negative (closer to zero) Pavlovian factors in OCD than HC (*t* = -2.039, *p =* 0.046) (Fig 4A). The main effect of group was also significant showing overall larger (that is, less negative) Pavlovian factors in OCD (*F*(1, 59) = 6.635, *p* = 0.013). These computational results showed an overall weaker influence of Pavlovian stimuli on instrumental behaviors in OCD patients.

To assess whether the Pavlovian factors related to OCD symptom severity, a partial correlation analysis between Y-BOCS scores and the Pavlovian factors across all subjects from both groups was conducted, controlling for anxiety and depression as measured by STAI and BDI. The CS- Pavlovian factor was positively related to Y-BOCS score (*r* = 0.430, *p =* 0.001) (Fig 4B) whereas the CS+ and CS0 Pavlovian factors were not related to Y-BOCS score (CS+: *r* = -0.105, *p* = 0.439, CS0: *r* = -0.073, *p* = 0.592).

### Specificity to OC symptoms

Because previous studies have reported abnormal PIT effects in depressed and anxious populations [5,9], we tested whether the reduced PIT effect in OCD patients was affected by the patients’ level of depression and anxiety. We performed a 2 (Group: HC, OCD) × 3 (CS: CS+, CS0, CS-) ANCOVA controlling for scores on STAI and BDI and compared the results with those from the ANOVA reported above. The ANCOVA revealed a significant main effect of CS (*F*(1.671, 91.906) = 7.002, *p* = 0.003, Greenhouse-Geisser corrected), a main effect of group (*F*(1,55) = 4.331, *p* = 0.042) and significant group × CS interaction (*F*(1.671, 91.906) = 12.095, *p* < 0.001, Greenhouse-Geisser corrected). It also revealed a significantly more positive CS- Pavlovian factor in the OCD than the HC group (CS-, *t* = 4.104, *p <* 0.001) and no effect of the CS0 Pavlovian factor (CS0, *t* = -1.559, *p =* 0.125). These results were consistent with the ANOVA results. However, in the ANCOVA the group difference for the CS+ Pavlovian factor failed to reach significance (CS+, *t* = -1.662, *p =* 0.102). These results showed the between-group difference in the CS- condition was present independent of depression and anxiety, whereas the between-group difference in the CS+ condition was not statistically independent of depression and anxiety.

## Discussion

This study is the first to examine PIT in OCD patients with a formal clinical diagnosis, and the first to use computational modeling to identify specific learning parameters accounting for PIT performance in OCD. Both OCD patients and HC performed a classic PIT paradigm. Overall, we found a reduction in PIT in the OCD group compared with HC. The weaker Pavlovian influence was especially apparent in the CS- condition in which OCD patients failed to inhibit instrumental responses in the presence of punishment stimuli.

In order to identify specific parameters that might explain this reduction in PIT, we performed computational modeling using modified Rescorla-Wagner models[8]. We compared models with different parameters and found that the model that best captured behavioral features during instrumental training was one that included separate learning rates for reward and punishment feedback, a single sensitivity measure for reinforcement, and accounted for bias towards the action of collecting the shell. This model revealed no between-group differences in the instrumental training stage suggesting that the between-group differences in the PIT stage resulted purely from the added influence from the Pavlovian system. The probabilities of particular actions in the PIT stage were generated by a linear combination of the instrumental action weights (including the expected instrumental values and the response bias) estimated in the instrumental stage and the Pavlovian factors estimated from the behavioral data in the PIT stage. The results showed the absolute values of Pavlovian factors were smaller in the OCD group corresponding to overall weaker Pavlovian influence on instrumental behavior. Moreover, these parameters were also related to OCD severity: OCD symptoms were positively correlated with the negative Pavlovian cue factors for the CS- in the PIT phase.

### PIT heterogeneity across compulsive disorders

The reduced PIT in OCD we observed is in contrast with another group of disorders characterized by compulsion, substance use disorders (SUDs), which are associated with increased PIT[29,39]. This difference might stem from the different mechanisms underlying compulsions in OCD and SUDs. Substance and behavioral addictions such as pathological gambling are often preceded by feelings of ‘pleasure, gratification, or relief at the time of committing the act’, which is ego-syntonic in nature[43]. However, compulsive behaviors in OCD patients are often completed to neutralize thoughts and relieve the anxiety related to obsessions[43]. Our results were consistent with these results as OCD patients in our study could not inhibit their behavior when facing punishment-predictive cues, which may result in departing from ego-syntonic behaviors.

These differences between OCD and SUDs in how compulsivity is expressed may be related to differences in recruitment of neural systems between the two groups. A neuroimaging study by Meunier and colleagues (2012) found differences in neural connectivity patterns between OCD and SUDs. Functional connectivity of orbitofrontal cortex was abnormally reduced in both OCD and stimulant-dependent individuals, but the degree of abnormality was greater among OCD patients, especially for orbitofrontal–dorsomedial connectivity [44]. This reduction in connectivity may be related to the ritualistic or manneristic movements associated with OCD[44], as the orbitofrontal–dorsomedial system is important for motor planning of goal-directed actions and adjustment according to reinforcement[45,46]. The finding that this system is less affected in SUDs than OCD is consistent with compulsivity influencing behavior in SUDs in a different manner, to wit, potentially via stronger Pavlovian influences on instrumental behaviors related to craving for appetitive consequences (e.g., the pleasant sensation after drug in-take) in SUDs. Overall, the different manifestations of compulsity in OCD and SUDs might be due to differences in adjusting behaviors based on expected consequences, which may in turn be associated with different patterns of recruitment of frontal control systems, especially within the orbitofrontal-dorsomedial system.

An important limitation of this study that could help to explain the differences between OCD and SUDs is that our study did not examine all types of Pavlovian cues that may be relevant in OCD. This study also utilized only previously neutral Pavlovian cues (e.g., fractal images) in order to enhance comparability with previous research. OCD patients may be more sensitive to cues that are related to their domain of compulsion. Previous research in substance use disorders has found that addiction-related cues can affect performance differently from other Pavlovian cues [30]. Future research could examine PIT with stimuli tailored to the individual person’s area of compulsion; for example, for patients with cleaning compulsions one could utilize cues indicating contamination (a dirty sink) or lack of contamination (a sanitized sink).

### Relationship between PIT and goal-directed instrumental learning in OCD

Another possible mechanism that could underlie reduced PIT in OCD is impairments in goal-directed learning in OCD. Previous research has revealed an imbalance within two different instrumental learning systems, the goal-directed and habitual systems, such that habitual control is dominant in OCD and goal-directed learning is impaired. This imbalance has been hypothesized as a fundamental mechanism underlying compulsive behaviors in OCD [1]. Behavioral research has shown that OCD patients are insensitive to reinforcer devaluation and continue to perform previously learned responses even after they are no longer rewarded, indicating habitual control of behavior [47]. For example, Gillan and colleagues asked OCD patients and healthy controls to perform a shock avoidance task in which subjects needed to produce correct responses (i.e., press left or right pedal) according to stimuli to avoid shock [48]. After overtraining, OCD patients presented stronger urge to make avoidance responses, even when they were informed the shock electrodes were detached[48]. This tendency to rely on habitual control was observed in both avoidance (avoiding shocks) and appetitive (earning points) tasks [49–51]. Theoretically, a shift from goal-directed to habitual control can occur for one or more of three reasons: increased strength of habitual learning, decreased strength of goal-directed learning, or a change in an independent arbitration process toward greater weighting of habits over goals [52]. Neuroimaging studies examining functional connectivity in OCD consistently report reduced connectivity within areas known to support goal-directed behavior[53], and between the goal-directed and habit systems[54,55], but no changes in connectivity within areas supporting the habitual system [54]. Finally, a task-based fMRI study using a symptom-provocation paradigm found reduced neural activation in brain regions implicated in goal-directed behavioral control (i.e., vmPFC and caudate nucleus) and increased activation in regions involved in habit learning (i.e., pre-supplementary motor area and putamen) [56]. Overall, evidence supports impairment in goal-directed processing in OCD attributable to changes within the goal-directed system itself alone or in concert with or changes in arbitration processes such that habitual control is chosen over goal-directed control.

Taken together, the reduction in PIT in OCD and known impairment in goal-directed behavior in OCD have an intriguing similarity with the declarative theory of PIT [10,25,26], which argues that PIT is supported by goal-directed processes. However, these results should be interpreted with caution. First, PIT was not designed as an assessment of goal directed behavior and the reduced levels of PIT in OCD cannot be taken as diagnostic of impaired goal directed behavior. Second, the types of evidence used to identify impaired goal-directed behavior in OCD are not exactly the same as those that were used to establish the goal directed nature of PIT in healthy adults. Goal-directed behaviors can reflect a variety of strategies, executive functions, and metacognitive beliefs, and it is unclear which of these many possibilities accounts for the reduced PIT in OCD. One possible strategy proposed in the declarative theory is that healthy people use the Pavlovian cues as contextual information that indicates the possibility of gaining reward for performing the instrumental behavior[57]. On this account, reduced PIT in OCD could be due to patients being less able to use the contextual information supplied by the Pavlovian cues to modulate their behavior. However, one challenge to the declarative view of PIT is that PIT can be an irrational behavior: without explicit knowledge that the Pavlovian cue is valid any changes in behavior may not actually lead to increased reward. In fact, to the degree that the Pavlovian cues indicate a conflicting behavior (e.g., CS+ combined with a bad shell can lead to incorrectly collecting the shell) they can actually lead to worse performance. One interpretation of the results could be that OCD patients are rational in making less use of the Pavlovian cues in determining their behavior; this may indicate a metacognitive difference between OCD and healthy controls in selection and use of which information to guide behavior rather than an impairment in executive function components of goal-directed behavior. Future studies could manipulate the elements of goal-directed behavior (i.e., action-outcome contingency or motivation of outcomes, manipulations of executive functions) to establish specific connections between goal-directed policies and PIT. Further studies could also examine general PIT, rather than the specific PIT examined in this study. General PIT, in which the rewards differ in the two tasks (e.g., monetary reward in one task, food reward in the other), is thought to have a greater reliance on automatic and motivational processes that are independent of awareness and do not rely on goal-directed cognitive resources [58,59]. Future research could also examine general PIT in OCD to understand whether this class of Pavlovian effects is affected in OCD.

### Role of negative cues and inhibitory control functions in PIT in OCD

Compared to healthy controls, OCD patients exhibited a reduced Pavlovian effect that was strongest when examining the influence of negative cues (CS-) on instrumental behaviors. The difference in the effect of CS+ on instrumental behaviors between OCD and HC was marginal in the original group analysis and did not reach significance in the ANCOVA controlling for level of depression and anxiety. That OCD patients show group differences from HC predominantly for negative cues could reflect an inherent difference in attention to negative cues and ability to use negative cues to affect behavior. Alternatively, differences in inhibitory control requirements for positive and negative Pavlovian cues may explain the difference. When the negative cue is present participants must reduce their responding (similar to a no-go task, which requires inhibition). In contrast, when the positive cue is present participants must increase responding (similar to a go task), which does not require inhibition. This asymmetry between the effects of reward and punishment in go versus no-go learning has been shown to be present in OCD: patients were especially poor at learning to withhold responding in punishment conditions [60]. Impaired inhibitory function is one of the most striking cognitive deficits in OCD [1,61,62].

Abnormal inhibition in OCD is apparent in the symptoms of OCD: OCD is characterized by intrusive, troubling thoughts that are perceived as the product of one’s mind and/or repetitive, compulsive behaviors or mental rituals. OCD patients also show impaired inhibition on a wide range of cognitive-behavioral tasks including motor inhibition as evidenced by commission errors on go/no-go tasks [63] and longer stop-signal reaction times [64]. An fMRI study found more activation in the left presupplementary motor area during successful inhibition accompanied by decreased activation in the right inferior parietal cortex and inferior frontal gyrus in OCD relative to controls, indicating atypical neural systems recruited for inhibition [65]. Impaired inhibitory processes in OCD are associated with reduced grey matter in orbitofrontal and right inferior frontal regions and increased grey matter in cingulate, parietal, and striatal regions[64].

## Conclusion

To summarize, at the behavioral level, OCD patients exhibited a weaker PIT effect than HC, especially under negative Pavlovian conditions. Computational modeling further found that Pavlovian factors were close to zero in OCD patients showing weaker Pavlovian influences on instrumental behaviors. Our results indicated that patients with OCD may be unable to use the contextual information provided by Pavlovian cues to alter their performance, particularly when they are required to inhibit performance. A greater emphasis on contextual factors may prove useful in developing therapies for those with OCD.
